# Prediction of Pathological Upgrading at Radical Prostatectomy in Prostate Cancer Eligible for Active Surveillance: A Texture Features and Machine Learning-Based Analysis of Apparent Diffusion Coefficient Maps

**DOI:** 10.3389/fonc.2020.604266

**Published:** 2021-02-04

**Authors:** Jinke Xie, Basen Li, Xiangde Min, Peipei Zhang, Chanyuan Fan, Qiubai Li, Liang Wang

**Affiliations:** ^1^ Department of Radiology, Tongji Hospital, Tongji Medical College, Huazhong University of Science and Technology, Wuhan, China; ^2^ Department of Radiology, University of Texas Southwestern Medical Center at Dallas, Dallas, TX, United States

**Keywords:** magnetic resonance imaging, prostatic neoplasms, Gleason score, active surveillance, machine learning

## Abstract

**Objective:**

To evaluate a combination of texture features and machine learning-based analysis of apparent diffusion coefficient (ADC) maps for the prediction of Grade Group (GG) upgrading in Gleason score (GS) ≤6 prostate cancer (PCa) (GG1) and GS 3 + 4 PCa (GG2).

**Materials and methods:**

Fifty-nine patients who were biopsy-proven to have GG1 or GG2 and underwent MRI examination with the same MRI scanner prior to transrectal ultrasound (TRUS)-guided systemic biopsy were included. All these patients received radical prostatectomy to confirm the final GG. Patients were divided into training cohort and test cohort. 94 texture features were extracted from ADC maps for each patient. The independent sample t-test or Mann−Whitney U test was used to identify the texture features with statistically significant differences between GG upgrading group and GG non-upgrading group. Texture features of GG1 and GG2 were compared based on the final pathology of radical prostatectomy. We used the least absolute shrinkage and selection operator (LASSO) algorithm to filter features. Four supervised machine learning methods were employed. The prediction performance of each model was evaluated by area under the receiver operating characteristic curve (AUC). The statistical comparison between AUCs was performed.

**Results:**

Six texture features were selected for the machine learning models building. These texture features were significantly different between GG upgrading group and GG non-upgrading group (*P* < 0.05). The six features had no significant difference between GG1 and GG2 based on the final pathology of radical prostatectomy. All machine learning methods had satisfactory predictive efficacy. The diagnostic performance of nearest neighbor algorithm (NNA) and support vector machine (SVM) was better than random forests (RF) in the training cohort. The AUC, sensitivity, and specificity of NNA were 0.872 (95% CI: 0.750−0.994), 0.967, and 0.778, respectively. The AUC, sensitivity, and specificity of SVM were 0.861 (95%CI: 0.732−0.991), 1.000, and 0.722, respectively. There had no significant difference between AUCs in the test cohort.

**Conclusion:**

A combination of texture features and machine learning-based analysis of ADC maps could predict PCa GG upgrading from biopsy to radical prostatectomy non-invasively with satisfactory predictive efficacy.

## Introduction

Prostate cancer (PCa) is the second leading cancer expected to be diagnosed and the fifth leading cause of death in men worldwide (1). Among older males, PCa was the most common cancer globally (2). Based on prostate specific antigen (PSA), clinical stage, and biopsy Gleason score (GS), PCa is stratified into low-risk (GS 2 to 6), intermediate-risk (GS 7), and high-risk (GS 8 to 10) groups (3). Active surveillance (AS) is an effective strategy for patients with low-risk PCa (4, 5), while the management of intermediate-risk PCa is controversial. Traditionally, radical prostatectomy (RP) is the preferred treatment for patients with intermediate-risk PCa (3). Recently, increasing interest has been paid in expanding the indications for AS to intermediate-risk PCa (6, 7). In addition, studies indicate that GS 3 + 4 PCa shows better prognosis than GS 4 + 3 PCa (8, 9). A new PCa grading system was developed during the 2014 International Society of Urological Pathology (ISUP) Consensus Conference (10), distinguishing GS 3 + 4 PCa [Grade Group(GG)2] from GS 4 + 3 PCa (GG3) because of the different prognosis. Current guidelines and studies support AS for selected intermediate-risk patients such as GG2, meanwhile RP is mostly recommended for the patient of GG3 or higher (7, 11–14). AS of PCa depends on GG at biopsy, which has shown great promise in limiting overtreatment of GS ≤6 PCa (GG1) and GG2. Nevertheless, studies showed that patients with biopsy proven GG1 and GG2 could upgrade to GG3 or higher after RP (15–17). Therefore, in order to limit overtreatment and ameliorate the risk of PCa progression, it is crucial to predict whether biopsy-proven GG would upgrade after RP.

Magnetic resonance imaging (MRI) is currently recognized as the best imaging modality for the diagnosis of PCa (18). Apparent diffusion coefficient (ADC) maps derived from diffusion-weighted imaging (DWI) sequences are useful imaging markers to evaluate the aggressiveness of PCa non-invasively, and have repeatedly proven to be correlated with GS (19–21). Furthermore, ADC maps could differentiate GG2 from GG3 (22, 23). Moreover, studies showed that ADC maps had the value of predicting GS upgrading (24, 25). Texture feature analysis has become a growing field in PCa imaging research. It has the potential to extract additional quantitative data from medical imaging which could improve diagnostic accuracy and help personal decision-making like preventing overtreatment (26). These texture features may be helpful for predicting GG upgrading. In addition, machine learning methods with or without texture features are promising to expand the clinical role of prostate MRI, which could assess the aggressiveness of PCa (27, 28). Furthermore, machine learning methods showed advantage of predicting Gleason pattern 4 in PCa (29), which could be useful to predict GG upgrading. However, the potential value of the combination of texture features and machine learning-based analysis of ADC maps in predicting GG upgrading has not been fully investigated.

The aim of this study is to explore whether a combination of texture features and machine learning-based analysis of ADC maps could predict GG upgrading to GG3 or higher after RP in GG1 and GG2.

## Materials and Methods

### Patients Information

**Figure 1:**
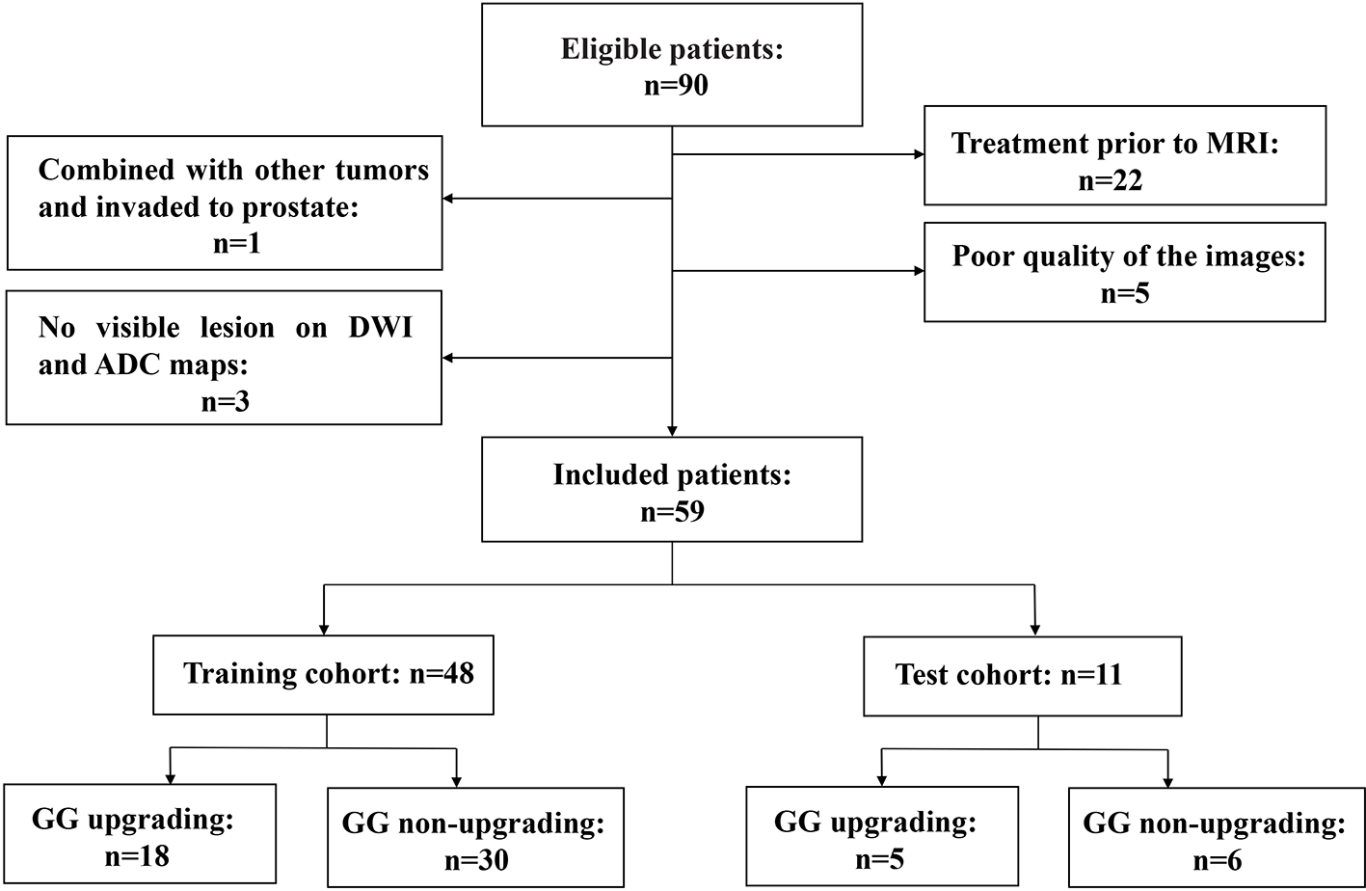
Flow chart of inclusion and exclusion criteria for patients.

This retrospective study was approved by our institutional review board. Studies have shown that GG will enable patients to understand their true risk stratification better and reduce overtreatment (30, 31). Therefore, between November 2016 and February 2020, consecutive patients who were biopsy-proven to have GG1 or GG2 and underwent MRI examination with the same MRI scanner prior to transrectal ultrasound (TRUS)-guided systemic biopsy were included in this study. All these patients received RP to confirm the final GG. MRI examination included a DWI sequence with 14 b-values (0–1500 s/mm^2^). The exclusion criteria were as follows: (a) patients with prior therapy for PCa; (b) poor quality of the images due to movement artifacts, magnetic susceptibility artifacts or the presence of hip implants; (c) no visible lesion on DWI and ADC maps; (d) combined with other tumors and invaded to prostate tissue such as bladder cancer and rectal cancer. The flow chart of inclusion and exclusion criteria for patients is shown in **Figure 1**. Ultimately, 59 patients were included in the final analysis. A study showed that most texture features extracted from ADC maps had no significant difference between GG1 and GG2 (32). Moreover, a previous study also delineated tumors on the histology with GS and classed as high-grade cancer if GS≥4 + 3 (GG3 or higher), or low-grade cancer if GS ≤3 + 4 (GG1 and GG2). They used texture feature to classify the high-grade cancer and low-grade cancer, which had satisfactory performance (33). Hence patients who were biopsy-proven to have GG1 or GG2 were divided into GG upgrading group and GG non-upgrading group according to whether they upgraded to GG3 or higher after RP. The included patients were divided into training cohort (n = 48) and test cohort (n = 11). Patients’ characteristics are shown in **Table 1**. The histopathologic analysis of the prostate after TRUS-guided systemic biopsy and RP was performed by experienced pathologists.

**Table 1:** Characteristics of patients.

Characteristics	Training cohort		Test cohort	
	GG upgrading	GG non-upgrading	GG upgrading	GG non-upgrading
Age (years)	65.33 ± 6.64	66.07 ± 6.71	68.40 ± 6.35	66.67 ± 6.09
PSA (ng/ml)	28.84 ± 30.77	20.78 ± 22.34	25.76 ± 21.08	13.91 ± 9.51
PSA, No.				
≤4 ng/ml	1	1	0	0
4−10 ng/ml	6	8	1	3
> 10 ng/ml	11	21	4	3
Biopsy GG, No.				
GG1 (3 + 3)	6	13	2	1
GG2 (3 + 4)	12	17	3	5
Prostate zone, No.				
Peripheral zone	13	13	3	3
Transitional zone	5	17	2	3
Tumor size(cm^3^)	8.09 ± 13.26	6.66 ± 10.27	5.70 ± 3.67	6.38 ± 2.60

PSA, prostate specific antigen; GG, Grade Group.

### MRI Examination

All patients underwent MRI examination with the same 3.0 T MRI scanner (Skyra, Siemens Healthcare, Erlangen, Germany), using an eighteen-channel abdomen coil and a spine phased-array coil. The scanning sequence included axial, coronal, sagittal T2-weighted turbo spin-echo sequence, axial T1-weighted turbo spin-echo sequence, and axial DWI. The minimum b value of axial DWI is 0 s/mm^2^, and the maximum b value is 1,500 s/mm^2^. The specific sequence parameters are shown in **Table 2**. The DWI images were fitted with mono-exponential model to automatically construct ADC maps by using the following formula:

$$S(b)/S_{0}=\exp(-b\cdot ADC)$$

**Table 2:** MRI sequence parameters.

Sequence	T1WI	T2WI	DWI
TR (ms)	807	6,500−6,880	4,300
TE (ms)	13	104	78
Thickness (mm)	5	3	3
Slice gap (mm)	0	0	0
Slices	26	22	22
FOV (mm^2^)	356 × 300	180 × 180	215 × 172
Matrix	320 × 240	384 × (307−356)	90 × 72
Flip angle (degree)	160°	160°	90°

TR, repetition time; TE, echo time; FOV, field of view; T1WI, T1-weighted imaging; T2WI, T2-weighted imaging; DWI, diffusion-weighted imaging.

S(b) is the signal intensity at a particular b value, and S_0_ is the signal intensity at b = 0 s/mm^2^. ADC is the diffusion coefficient of the mono-exponential model.

### Image Segmentation and Feature Extraction

**Figure 2:**
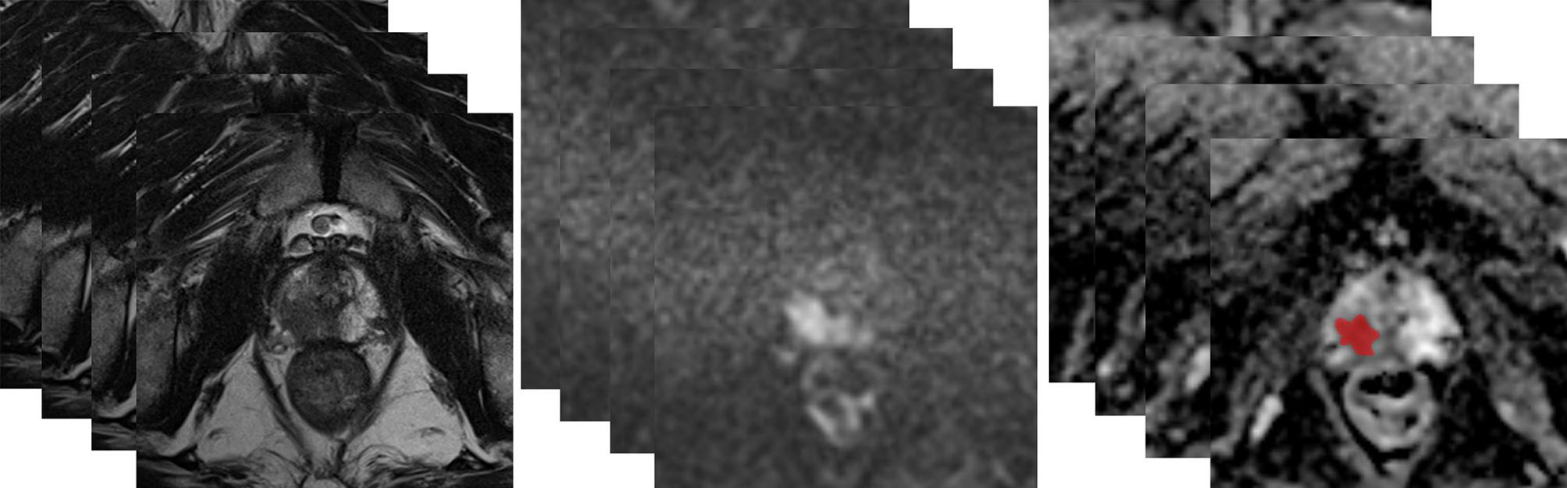
A 63-year-old man with a tumor of GS 3 + 4 (GG2) in the right peripheral zone was diagnosed at TRUS-guided 12-core systemic biopsy. The whole tumor was delineated by stacking up regions of interest slice by slice on the ADC maps.

MRI images of all patients were exported in DICOM format through the post-processing workstation. ADC maps were obtained for image analysis. The three-dimensional data analysis module of the MaZda software (version 4.6; http://eletel.eu/mazda) was used for manual segmentation. A proficient radiologist manually delineated the regions of interest covering the whole tumor slice by slice on ADC maps without knowing the pathological results to form volume of interest (VOI) (**Figure 2**). As the ADC maps were automatically reconstructed from DWI images, the ADC maps had the same locations as the DWI images. Besides, T2WI had high spatial resolution. The radiologist manually delineated VOI on ADC maps using T2WI (axial, coronal, sagittal) and DWI images as reference based on previous study (34). The same radiologist manually re-segmented the images a month later. A senior radiologist with 20 years of experience with prostate MRI verified all the segmentations.

The texture features of ADC maps were extracted by MaZda software, including nine histogram features, five absolute gradient features, 11 gray-level co-occurrence matrix features (GLCM), and five run-length matrix features (RLM). The GLCM were computed up to five times, for (d,0,0), (0,d,0), (d,d,0), (d,−d,0),(0,0,d), where the distance d take the value of 1. The RLM were computed five times for each VOI (for horizontal, vertical, 45-degree, 135-degree and Z directions). Therefore, a total of 94 features were extracted. All texture features were normalized by “ ± 3 sigma” option, which is equivalent to the range [*μ* − 3*σ*, *μ* + 3*σ*] where *μ* is the image mean and *σ* denotes its standard deviation (both *μ* and 3*σ* are computed separately for every VOI).

### Statistical Analysis

The texture feature selection and model construction in the training cohort was performed using following steps. Comparisons were performed between GG upgrading group and GG non-upgrading group. The intraclass correlation coefficient (ICC) was calculated to evaluate the test-retest reliability. The Kolmogorov-Smirnov test was used to evaluate whether the texture features conform to the normal distribution. The independent t-test was performed to compare texture features conforming to normal distribution for differentiating GG upgrading group from GG non-upgrading group. The Mann−Whitney U test was used for the texture features violating the normal distribution. As the least absolute shrinkage and selection operator (LASSO) regression model has satisfying performance for filtering features (35), it with five-fold cross-validation was adopted for further feature selection. Features with non-zero coefficients were selected. Texture features of GG1 and GG2 were compared based on the final pathology of radical prostatectomy.

For the features selected by the LASSO regression model, four supervised machine learning methods were employed. The machine learning methods were as follows: random forests (RF), decision tree (DT), support vector machine (SVM) and nearest neighbor algorithm (NNA). The receiver operating characteristic (ROC) curve was constructed in both the training cohort and test cohort. The area under the curve (AUC), sensitivity and specificity were used to evaluate the prediction performance of each machine learning model. The De.Long test was used for statistical comparison between AUCs (36).

Feature reduction, ICC and comparison of texture features between GG1 and GG2 were performed on SPSS software (version 21; www.ibm.com/software/analytics/spss). And R software (version 3.6.1; www.r-project.org) was used for LASSO logistic regression model, machine learning methods and ROC analysis. And MedCalc software (version 19.5.6; www.medcalc.org) was used for statistical comparison between AUCs. The following R packages were used: the “lars” package was used to perform the LASSO regression model; the “randomForest” package was used to perform RF; the “rpart” package was used to perform DT; the “e1071” package was used to perform SVM; the “kknn” package was used to perform NNA; and the “pROC” package was used to construct the ROC curve. *P <*0.05 was considered as statistically significant.

## Results

**Figure 3:**
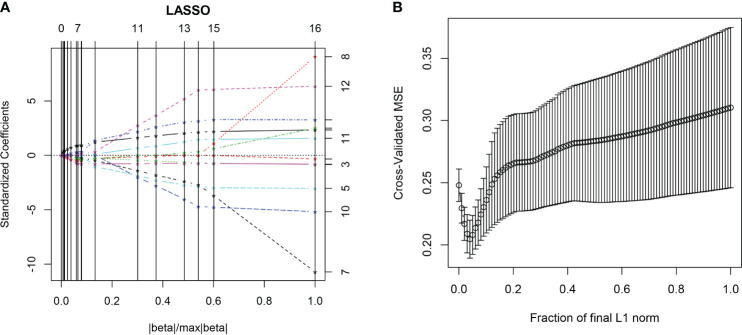
The LASSO algorithm. In the five-fold cross-validation (CV) of the LASSO algorithm, the coefficients of texture features change with parameters **(A)**. With the changes of CV, the model with minimum mean square error was selected **(B)**.

**Figure 4:**
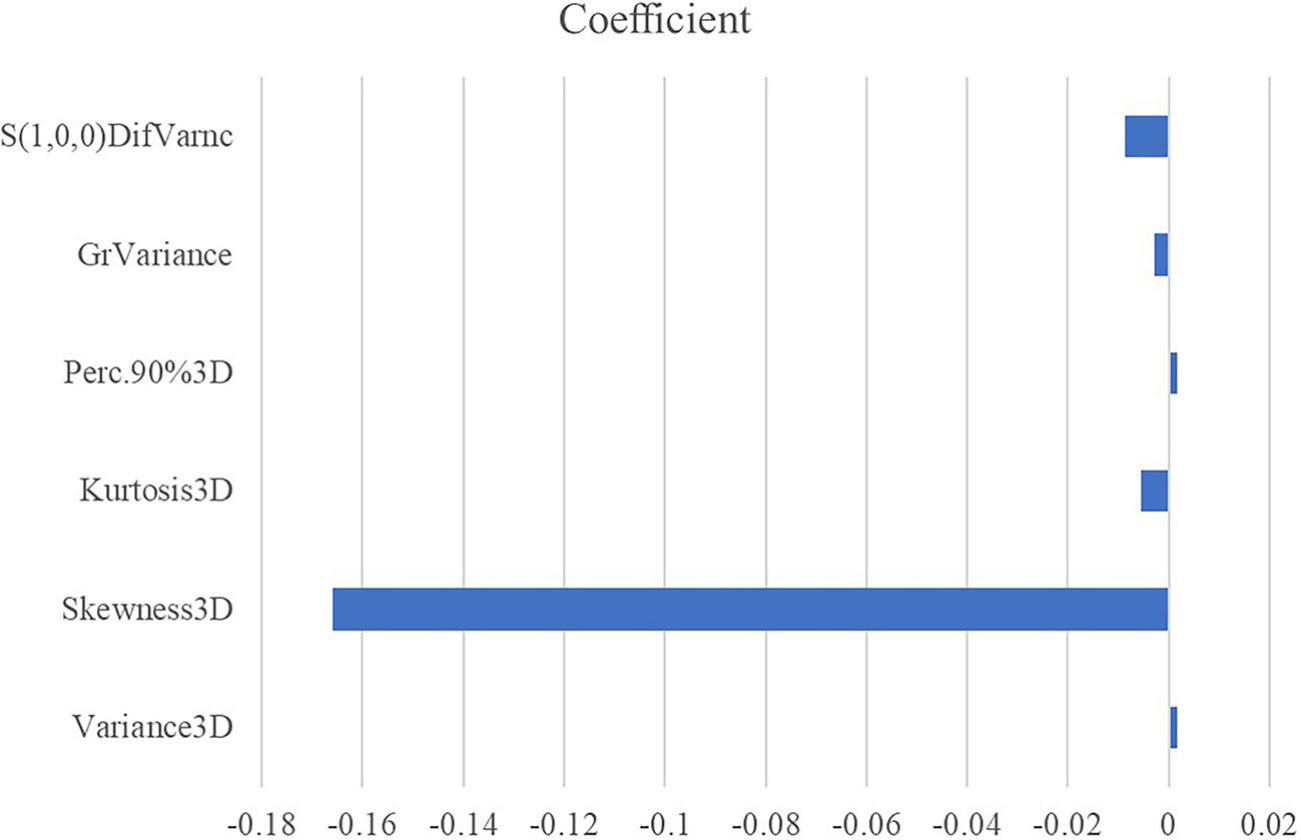
The absolute value of the coefficients from the LASSO algorithm represents the contribution of each feature.

**Figure 5:**
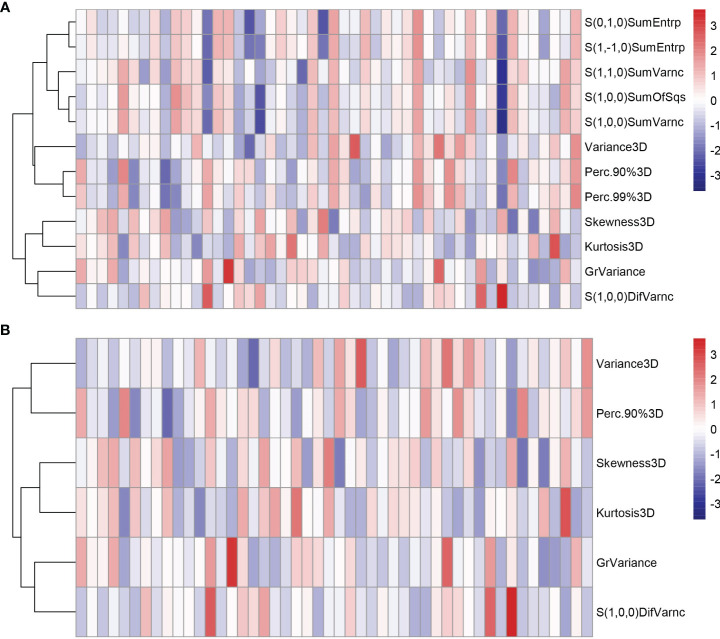
The heatmaps of texture features in the training cohort before **(A)** and after **(B)** LASSO algorithm. Some texture features were excluded after the LASSO algorithm, reducing the complexity of the model.

All the 94 texture features extracted from ADC maps have satisfactory test−retest reliability due to their ICC>0.8 (0.871−0.999). With the independent t-test and Mann−Whitney U test, 12 texture features were selected. Finally, through the five-fold cross-validation of the LASSO algorithm, six texture features (four histogram features, one absolute gradient feature, one GLCM) with non-zero coefficients were included to construct the machine learning models. The process of texture feature selection using the LASSO algorithm is shown in **Figure 3**. The six texture features include variance, skewness, kurtosis, 90% percentile, variance of absolute gradient (GrVariance) and S (1,0,0) difference variance [S(1,0,0)DifVarnc]. The contribution of each texture feature selected by the LASSO algorithm is shown in **Figure 4**. The LASSO algorithm reduced the complexity of the model. The heatmaps of texture features before and after LASSO algorithm are shown in **Figure 5**. The six texture features selected for constructing models had no significant difference between GG1 and GG2 in the training cohort and test cohort based on the final pathology of radical prostatectomy. The results of the comparison of texture features between GG1 and GG2 are shown in **Table 3**.

**Table 3:** Comparison of texture features between GG1 and GG2.

Texture feature		t	P
Training cohort	variance	0.222	0.826
	skewness	−1.05	0.300
	kurtosis	0.229	0.821
	90% percentile	0.951	0.350
	GrVariance	0.695	0.493
	S(1,0,0)DifVarnc	1.623	0.116
Test cohort	variance	0.952	0.395
	skewness	−0.599	0.582
	kurtosis	0.128	0.905
	90% percentile	−0.842	0.447
	GrVariance	0.655	0.548
	S(1,0,0)DifVarnc	−0.593	0.585

GrVariance, variance of absolute gradient; S(1,0,0) DifVarnc, S(1,0,0) difference variance; P < 0.05 was considered as statistically significant.

According to the statistical comparison between AUCs in the training cohort, the diagnostic performance of NNA and SVM was better than RF. The AUC, sensitivity, and specificity of NNA were 0.872 (95% CI: 0.750−0.994), 0.967, and 0.778, respectively. The AUC, sensitivity, and specificity of SVM were 0.861 (95%CI: 0.732−0.991), 1.000, and 0.722, respectively. Although the performance of RF was not as good as NNA and SVM, its performance is also satisfactory with the AUC, sensitivity, and specificity of 0.728 (95%CI: 0.569−0.887), 0.900 and 0.556, respectively. Moreover, the LASSO regression model in the training cohort also had satisfactory predictive efficacy, whose AUC, sensitivity and specificity were 0.820 (95% CI: 0.680−0.960), 0.933, and 0.722, respectively. Despite the predictive efficacy of the models in the test cohort was not as satisfactory as the training group, it was acceptable (37). There was no significant difference between AUCs in the test cohort. The ROC curves of machine learning models and LASSO regression model for discriminating GG upgrading from GG non-upgrading in the training cohort and test cohort are shown in **Table 4**. The results of statistical comparison between AUCs are shown in **Table 5**.

**Table 4:** Predictive performance of each model.

Model		AUC (95% CI)	Sensitivity	Specificity
Training cohort	LASSO	0.820 (0.680–0.960)	0.933	0.722
	RF	0.728 (0.569–0.887)	0.900	0.556
	DT	0.822 (0.701–0.994)	0.700	0.944
	SVM	0.861 (0.732–0.991)	1.000	0.722
	NNA	0.872 (0.750–0.994)	0.967	0.778
Test cohort	LASSO	0.667 (0.283–1.000)	1.000	0.600
	RF	0.633 (0.288–0.979)	0.667	0.600
	DT	0.667 (0.336–0.997)	0.333	1.000
	SVM	0.617 (0.266–0.968)	0.833	0.400
	NNA	0.717 (0.391–1.000)	0.833	0.600

AUC, area under the ROC curve; ROC, receiver operating characteristic curve; 95% CI, 95% confidence interval; LASSO, least absolute shrinkage and selection operator regression model; RF, random forests; DT, decision tree; SVM, support vector machine; NNA, nearest neighbor algorithm.

**Table 5:** Pairwise comparison of AUCs.

Model		Z statistic	P
Training cohort	RF~DT	1.217	0.2237
	RF~SVM	2.512	0.0120*
	RF~NNA	1.987	0.0470*
	DT~SVM	0.589	0.5555
	DT~NNA	0.683	0.4947
	SVM~NNA	0.169	0.8657
	RF~LASSO	1.540	0.1235
	DT~LASSO	0.0262	0.9791
	SVM~LASSO	1.062	0.2880
	NNA~LASSO	0.642	0.5208
Test cohort	RF~DT	0.206	0.8366
	RF~SVM	0.128	0.8981
	RF~NNA	0.378	0.7055
	DT~SVM	0.302	0.7630
	DT~NNA	0.238	0.8119
	SVM~NNA	0.612	0.5403
	RF~LASSO	0.249	0.8032
	DT~LASSO	0.000	1.000
	SVM~LASSO	0.277	0.7819
	NNA~LASSO	0.225	0.8218

RF, random forests; DT, decision tree; SVM, support vector machine; NNA, nearest neighbor algorithm; LASSO, least absolute shrinkage and selection operator regression model. P < 0.05 was considered as statistically significant.

## Discussion

Predicting PCa GG upgrading from biopsy to RP non-invasively is crucial for PCa management and prognosis. The Prostate Imaging-Reporting and Data System (PI-RADS) and PSA were used to evaluate PCa aggressiveness and GS upgrading. Despite being widely applied, the unavoidable inter-reader variability of PI-RADS and the low accuracy of PSA may lead inappropriate management (38, 39). ADC maps and texture features extracted from original medical images can provide more quantitative and functional information of tumor characteristics. Moreover, machine learning methods can construct classifiers with good predictive efficacy. The combination of these two techniques has potential to predict GG upgrading and help select appropriate therapeutic strategy. At present, there is no research that combines texture features and machine learning methods to predict GG upgrading.

Our study showed that the combination of texture features and machine learning methods based on ADC maps yielded satisfactory predictive efficacy. It is a valuable way to decide whether to adopt AS or not for patients with biopsy proven GG1 or GG2, which could be widely used in clinical practice. The diagnostic performance of NNA and SVM was better than RF in the training cohort. The predictive efficacy of the models in the test cohort was not as satisfactory as the training cohort, possibly because the sample size of the test cohort was small. Park S Y et al. suggested that DWI may help predict GS upgrading in PCa with biopsy-proven GS ≤6, and the AUCs of the DWI variables such as mean ADC for predicting GS upgrading were 0.711–0.760 (24). Another study presented a multiparametric MRI (mp-MRI)-based radiomics approach to accurately predict upgrading in GS, and the study indicated that the radiomics signature on ADC showed the best predictive performance with the AUC of 0.805 (40).

Texture features can provide large amounts of quantitative and objective information from original medical images that are easily ignored by naked eye observation and help to select clinically relevant biomarkers for disease evaluation. In current study, six texture features including four histogram features, one absolute gradient feature and one GLCM were selected as optimal features to construct predictive model. All the six texture features including variance, skewness, kurtosis, 90% percentile, GrVariance and S(1,0,0)DifVarnc indicated the heterogeneity of PCa. The different values of all the selected texture features between GG upgrading group and GG non-upgrading group showed the different aggressiveness of PCa. These texture features could show subtle changes in tissue patterns more clearly. This result was consistent with previous studies (41, 42). Nevertheless, there is also a controversy that ADC texture features are limited for the prediction of GS upgrading (43). Therefore, our study combines texture features and machine learning methods to improve the predictive performance.

In current study, the combination of texture features and machine learning based on ADC maps was performed to provide tissue information and analyze GG upgrading. Machine learning analysis based on varied biomarkers has been successfully applied in PCa detection and evaluation (39, 44–46). In the study by Nitta S et al., the age of the patients, PSA level, prostate volumes, and white blood cell count in urinalysis were used as input data for the machine learning methods, reaching the higher AUCs than the AUCs of the PSA level, PSA density and PSA velocity (39). Li J et al. combined SVM and features derived from mp-MRI applied for automatic classification of PCa with an AUC of 0.99 (44). Liu B et al. found that the dynamic contrast-enhanced (DCE)-MRI original image-derived features integrated with machine learning methods could predict PCa invasiveness non-invasively with high accuracy (45). In a recent study by Winkel D J et al., using quantitative imaging parameters as input, machine learning models outperformed PI-RADS assessment scores in the prediction of PCa (46). All these studies indicated that machine learning methods could help to evaluate the heterogeneity and aggressiveness of PCa. However, there is a lack of research about the prediction of GG upgrading using the combination of texture features and machine learning methods. Compared with other studies, our study focused on predicting GG upgrading rather than distinguishing between malignant and normal prostate region. In our study, the PCa lesions were delineated slice by slice, and texture features provided a large number of valuable tissue information combined with machine learning methods obtained satisfactory predictive performance (the AUCs of 0.728−0.872).

Our study has several limitations. Firstly, our analysis was performed on a retrospective analysis of a small group of patients which is from a single center. More data from multiple centers are needed to validate our results. Secondly, due to the fact that some tumors simultaneously invaded the peripheral zone (PZ) and the transitional zone (TZ), and a small number of patients were included, PZ and TZ tumors were not investigated separately. In the future work, larger samples are needed to analyze PZ and TZ tumors separately. Thirdly, patients without visible lesions on ADC maps were excluded in this study, because we were unable to delineate the tumor region during MRI segmentation. This could lead to some selection bias. Despite the limitations of our study, we believe that the principal results of our preliminary study are sufficiently valid.

In conclusion, in our study, we established four machine learning models based on the texture features extracted from ADC maps to predict PCa GG upgrading from biopsy to RP non-invasively which had satisfactory predictive efficacy. Further studies are warranted to validate and confirm our primary findings. These machine learning models may have the potential to be an effective complement to conventional MRI and help clinicians select appropriate therapeutic strategy.

## Data Availability Statement

The raw data supporting the conclusions of this article will be made available by the authors, without undue reservation.

## Ethics Statement

The studies involving human participants were reviewed and approved by Tongji Hospital, Tongji Medical College, Huazhong University of Science and Technology institutional review board. The patients provided their written informed consent to participate in this study.

## Author Contributions

LW and JX contributed to conception and design of the study. JX, PZ, and CF acquired of data. JX and LW analyzed and interpreted data. JX and XM performed the statistical analysis. LW and BL supervised the work. JX wrote the first draft of the manuscript. LW, QL, BL, and XM revised article critically for important intellectual content. All authors contributed to the article and approved the submitted version.

## Funding

This research was supported by the National Natural Science Foundation of China (Grant numbers: 81171307, 81671656).

## Conflict of Interest

The authors declare that the research was conducted in the absence of any commercial or financial relationships that could be construed as a potential conflict of interest.
